# 

**Published:** 1888-02

**Authors:** 


### The Chicago Medical Journal and Examiner.

Volume 56. Number 2.

### CONTENTS OF THIS NUMBER.

**Original Communications—** PAGE.

Primary Myoma of the Broad Ligament. Bayard Holmes ........ 65
Cyst of the Pancreas. C. Fenger and A. Holmboe ........ 74

**Illinois State Board of Health—**

Annual Meeting ........ 78

**Medical Societies—**

BALTIMORE GYNÆCOLOGICAL AND OBSTETRICAL SOCIETY—

Tetanoid Constriction of the Uterus. H. M. Wilson ........ 88
Laparotomy, with Report of Three Cases. H. P. C. Wilson ........ 89
A Case of Nyphomania. W. P. Chunn ........ 94
A New Uterine Curette. T. A. Ashby ........ 95

ROYAL ACADEMY OF MEDICINE IN IRELAND—

PATHOLOGICAL SECTION—

Hour-Glass Contraction of the Stomach. Dr. Finny ........ 96
Phlegmasia Alba Dolens. Professor Bennett ........ 97

CHICAGO MEDICAL SOCIETY—Stated Meeting November 7th—

Pertussis. J. A. Robison ........ 99
Vaginal Hysterectomy J H. Etheridge ........ 100
Nephrectomy. C. T. Parkes ........ 100

NEW YORK ACADEMY OF MEDICINE—

Hernia. T. H. Burchard ........ 102

PATHOLOGICAL SOCIETY OF PHILADELPHIA—

Malarial Germ of Lavaran. W. T. Councilman ........ 105

THE INTERCOLONIAL MEDICAL CONGRESS OF AUSTRALASIA—

The President's Address ........ 107
Section of Medicine ........ 108
Section of Surgery ........ 109
Section of Gynæcology ........ 109
Section of State Medicine ........ 109

THE GERMAN SOCIETY OF NATURALISTS AND PHYSICIANS—

Natural Science and Schools ........ 111
Plant-Life and Plant-Respiration ........ 112
Putrefaction and Infectious Diseases ........ 112

EDINBURGH MEDICO-CHIRURGICAL SOCIETY—

The Ætiology of Tumors. J. Smith ........ 114

THE MEDICAL SOCIETY OF LONDON—

Filiaria Sanguinis Hominis. S. MacKenzie ........ 115
Cardiac Failure in Pulmonary Obstruction. Dr. Thorowood ........ 115

**Extracts and Abstracts—**

A Curious Disease called "Head-Drop" ........ 116
Saccharin ........ 118
Saccharine "Tabloids" ........ 119
"Cholera at New York" ........ 120
The Bacillus of Scarlatina ........ 121
Stellate and Trabecular Keratitis ........ 122
"A Cure for Wrinkles" ........ 122
Jaborandi as a Galactophore ........ 123
"New Uterine Support for Horse-Women" ........ 123

**Books Received—**

**Pamphlets Received—**

Items—

